# Slowing of Motor Nerve Conduction Velocity in Streptozotocin-induced Diabetic Rats is Preceded by Impaired Vasodilation in Arterioles that Overlie the Sciatic Nerve

**DOI:** 10.1155/EDR.2000.131

**Published:** 2000

**Authors:** Lawrence J. Coppey, Eric P. Davidson, Joyce A. Dunlap, Donald D. Lund, Mark A. Yorek

**Affiliations:** 1 Veterans Affairs Medical Center Diabetes Endocrinology Research Center and Department of Internal Medicine University of Iowa Iowa IA 52246 USA; 2 3 E 17 Veterans Affairs Medical Center Iowa IA 52246 USA

## Abstract

Diabetes mellitus produces marked abnormalities in motor nerve conduction, but the mechanism is not clear. In the present study we hypothesized that in the streptozotocin (STZ)-induced diabetic rat impaired vasodilator function in arterioles that provide circulation to the region of the sciatic nerve is associated with reduced endoneural blood flow (EBF) and that these defects precede slowing of motor nerve conduction velocity, and thereby may contribute to nerve dysfunction. As early as three days after the induction of diabetes endoneural blood flow was reduced in the STZ-induced diabetic rat. Furthermore, after 1 week of diabetes acetylcholine- induced vasodilation was found to be impaired. This was accompanied by an increase in the superoxide level in arterioles that provide circulation to the region of the sciatic nerve as well as changes in the level of other markers of oxidative stress including an increase in serum levels of thiobarbituric acid reactive substances and a decrease in lens glutathione level. In contrast to the vascular related changes that occur within 1 week of diabetes, motor nerve conduction velocity and sciatic nerve Na^+^/k^+^ ATPase activity were significantly reduced following 2 and 4 weeks of diabetes, respectively. These studies demonstrate that changes in vascular function in the STZ-induced diabetic rat precede the slowing of motor nerve conduction velocity (MNCV) and are accompanied by an increase in superoxide levels in arterioles that provide circulation to the region of the sciatic nerve.


*Int. Jnl. Experimental Diab. Res.*, Vol. 1, pp. 131–143
Reprints available directly from the publisher
Photocopying permitted by license only

© 2000 OPA (Overseas Publishers Association) N.V.
Published by license under
the Harwood Academic Publishers imprint,
part of The Gordon and Breach Publishing Group.
Printed in Malaysia.

### Slowing of Motor Nerve Conduction Velocity in Streptozotocin-induced Diabetic Rats is Preceded by Impaired Vasodilation in Arterioles that Overlie the Sciatic Nerve

LAWRENCE J. COPPEY, ERIC P. DAVIDSON, JOYCE A. DUNLAP, DONALD D. LUND and MARK A. YOREK*

*Veterans Affairs Medical Center, Diabetes Endocrinology Research Center and Department of Internal Medicine, University of Iowa, Iowa City, IA, 52246*

*(Received in final form 14 February 2000)*

**Diabetes mellitus produces marked abnormalities in motor nerve conduction, but the mechanism is not clear. In the present study we hypothesized that in the streptozotocin (STZ)-induced diabetic rat impaired vasodilator function in arterioles that provide circulation to the region of the sciatic nerve is associated with reduced endoneural blood flow (EBF) and that these defects precede slowing of motor nerve conduction velocity, and thereby may contribute to nerve dysfunction. As early as three days after the induction of diabetes endoneural blood flow was reduced in the STZ-induced diabetic rat. Furthermore, after 1 week of diabetes acetylcholine-induced vasodilation was found to be impaired. This was accompanied by an increase in the superoxide level in arterioles that provide circulation to the region of the sciatic nerve as well as changes in the level of other markers of oxidative stress including an increase in serum levels of thiobarbituric acid reactive substances and a decrease in lens glutathione level. In contrast to the vascular related changes that occur within 1 week of diabetes, motor nerve conduction velocity and sciatic nerve $Na^+/K^+$ ATPase activity were significantly reduced following 2 and 4 weeks of diabetes, respectively. These studies demonstrate that changes in vascular function in the STZ-induced diabetic rat precede the slowing of motor nerve conduction velocity (MNCV) and are accompanied by an increase in superoxide levels in arterioles that provide circulation to the region of the sciatic nerve.**

*Keywords:* Diabetes, vasodilation, diabetic neuropathy, acetylcholine, oxygen radicals, endothelium

### INTRODUCTION

The purpose of this study was to examine the relationship between diabetes-induced reduction in sciatic nerve motor nerve conduction velocity, endoneural blood flow and vascular reactivity of arterioles that provide circulation to the region of the sciatic nerve using the streptozotocin-induced diabetic rat model.

*Corresponding author. 3 E 17 Veterans Affairs Medical Center, Iowa City, IA 52246. Tel.: (319) 338-0581 ext. 7630, Fax: (319) 339-7025, e-mail: myorek@icva.gov

In a previous study we showed that acetylcholine-induced endothelium-dependent vasodilation of arterioles that provide blood flow to the region of the sciatic nerve is impaired by diabetes.[1] This defect was associated with a decrease in neuronal blood flow of the sciatic nerve and slowing of motor nerve conduction velocity. In the present study we investigated whether the impaired vasodilation of these arterioles in diabetic rats preceded the changes in endoneural blood flow and nerve conduction.

Diabetes mellitus produces marked abnormalities in motor nerve conduction, but the mechanism is not clear. Cameron *et al.* reported that hyperglycemia-induced blood flow reduction and resultant endoneural hypoxia are important factors underlying nerve conduction deficits early in the development of diabetic neuropathy.[2] In their studies, they showed that nerve blood flow was reduced as early as 1 week after diabetes induction. In other studies conducted with older animals reductions in nerve blood flow were apparent and preceded the decrease in nerve conduction velocities.[3] In the present study we examined the relationship between changes in endoneural blood flow and nerve conduction velocity induced by diabetes, and the impairment in acetylcholine-induced vasodilation in arterioles that provide circulation to the region of the sciatic nerve.

The effect of diabetes on vascular reactivity has been studied in a variety of vascular tissues. In the aorta from several species, agonist-induced endothelium-dependent vasodilation is impaired by diabetes and by acute hyperglycemia.[4–7] Similar alterations in vasodilator responses have been observed in mesenteric arteries from diabetic rats.[8–11] Other studies have shown reduced acetylcholine-induced vasodilation of the basilar artery,[12, 13] renal interlobar artery, [14] and coronary artery [15, 16] in diabetes. Therefore, impaired endothelial-dependent vasodilation of the aorta and arteries supplying the kidney, heart and as reported recently by us, the region of the sciatic nerve, is a common defect in diabetes. In this study we report that a reduction in endoneural blood flow in the sciatic nerve occurs as early as three days after the induction of diabetes. Acetylcholine-induced vasodilation by arterioles that provide circulation to the region of the sciatic nerve is also impaired early in diabetes and is accompanied by an increase in superoxide levels in these vessels. These changes precede the slowing of motor nerve conduction velocity and the reduction in $Na^+/K^+$ ATPase activity in the sciatic nerve.

### METHODS

### Animals

Male Sprague–Dawley (Harlan Sprague Dawley, Indianapolis, IN) rats 8–9 weeks of age were used for these studies. The animals were housed in a certified animal care facility and food and water were provided ad libitum. All institutional and NIH guidelines for use of animals were followed. Diabetes was induced by intravenously injecting streptozotocin (60 mg $kg^{-1}$ in 0.9% NaCl, adjusted to a pH 4.0 with 0.2 M sodium citrate). Control rats were injected with vehicle alone. The rats were anesthetized with methoxyflurane before injection. Diabetes was verified 24 h later by evaluating blood glucose levels with the use of glucose oxidase reagent strips (Boehringer–Mannheim, Indianapolis, IN). Rats having blood glucose level of 300 mg/dl (16.7 mM) or greater were considered to be diabetic. Studies were conducted 3, 7, 14, 21 and 28 days after the induction of diabetes. On the day of the experiment rats were anesthetized with Nembutal i.p. (50 mg $kg^{-1}$, Abbott Laboratories, North Chicago, IL). Following the determination of motor nerve conduction velocity (MNCV) and endoneural blood flow (EBF), the abdominal aorta was isolated and occluded 1–2 cm above the branch of the common iliac artery. Distal to the occlusion a solution containing India ink with 2% gelatin [1, 17] was injected to facilitate the identification of the superior gluteal and internal pudendal arteries, which arise from the common iliac artery. The rat was then sacrificed by exsanguination, and body temperature lowered with topical ice. Samples of the left sciatic nerve were then taken for determination of $Na^+/K^+$ ATPase activity, sorbitol, fructose and myo-inositol content and conjugated diene level. The lens was also collected for determination of glutathione levels. Levels of thiobarbituric acid reactive substances (TBARS) in the serum and lactate/pyruvate ratio in the aorta were also determined. Samples for blood glucose measurements were also taken on the day of the experiment.

### Motor Nerve Conduction Velocity

MNCV was determined as previously described using a noninvasive procedure in the sciatic-posterior tibial conducting system in a temperature controlled environment. [1, 18] The left sciatic nerve was stimulated first at the sciatic notch and then at the Achilles tendon. Stimulation consisted of single 0.2 ms supra maximal (8V) pulses through a bipolar electrode (Grass S44 Stimulator, Grass Medical Instruments, Quincy MA). The evoked potentials were recorded from the interosseous muscle with a unipolar platinum electrode and displayed on a digital storage oscilloscope (model 54600A Hewlett Packard, Rolling Meadows, IL). MNCV was calculated by subtracting the distal from the proximal latency measured in milliseconds from the stimulus artifact of the take-off of the evoked potential and the difference was divided into the distance between the two stimulating electrodes measured in millimeters using a Vernier caliper. The MNCV was reported in meters per second.

### Endoneural Blood Flow

Immediately after determination of MNCV, sciatic endoneural nutritive blood flow was determined as described by Cameron *et al.* [2, 19] The trachea was intubated for artificial ventilation and a carotid cannula inserted to monitor mean arterial blood pressure. Core temperature was monitored using a rectal probe and temperature regulated between 36 and 37°C using a heating pad and radiant heat. The right sciatic nerve was carefully exposed by a small surgical incision and the surrounding skin sutured to a plastic ring. The isolated area was filled with mineral oil, at 37°C to a depth of 1 cm to minimize diffusion of hydrogen gas from the nerve. The rats were then artificially ventilated. A glass insulated platinum microelectrode (tip = 2 μm) was inserted into the sciatic nerve, proximal to the trifurcation, and polarized at 0.25 V with respect to a reference electrode inserted subcutaneously into the flank of the rat. Once the recording had stabilized the inspired air was modified to contain 10% hydrogen gas and this gas flow continued until the hydrogen current recorded by the electrode had stabilized, indicating equilibrium of the inspired air with arterial blood. The hydrogen gas supply was then discontinued and the hydrogen clearance curve recorded until a baseline was achieved. The hydrogen clearance data was fitted by computer to a mono- or bi-exponential curve using commercial software (Prism, GraphPad, San Diego, CA) and nutritive blood flow, (ml/min/100 g), calculated using the equation described by Young (1980) and vascular conductance, (ml/min/100 g/mm Hg) determined by dividing nutritive blood flow by the average mean arterial blood pressure. Two recordings were made for each rat at different locations along the nerve and the final blood flow value averaged.

### Vascular Reactivity

Videomicroscopy was used to investigate *in vitro* vasodilatory responsiveness of arterioles supplying the region of the sciatic nerve (branches of the superior gluteal and internal pudendal arteries) as previously described. [1] The vessels used for these studies were generally oriented longitudinally in relation to the sciatic nerve; however, on occasion radially oriented vessels were also used. No differences were observed in acetylcholine-induced vasodilation based on the orientation of the vessel to the sciatic nerve. Since vessels overlying the sciatic nerve were used in this study they should be regarded as epineurial rather than perineurial. To isolate these vessels the common iliac was exposed and the branch points of the internal pudendal and superior gluteal arteries identified. The vessels were then clamped, and tissue containing these vessels and its branches dissected en bloc. The block of tissue was immediately submerged in an cooled (4°C), oxygenated (20% $O_2$, 5% $CO_2$ and 75% $N_2$) Krebs Henseleit physiological saline solution (PSS) of the following composition (in mM): NaCl 118, KCl 4.7, $CaCl_2$ 2.5, $KH_2PO_4$ 1.2, $MgSO_4$ 1.2, $NaHCO_3$ 20, $Na_2EDTA$ 0.026, and 5.5 glucose for dissection. Branches of the superior gluteal and internal pudendal arteries (50 – 150 μm internal diameter and 2 mm in length) were carefully dissected and trimmed of fat and connective tissue. Both ends of the isolated vessel segment were cannulated with glass micropipettes filled with PSS (4°C), and secured with 10-0 nylon Ethilon monofilament sutures (Ethicon, Inc., Cornelia, GA). The pipettes were attached to a single pressure reservoir (initially set at 20 mm Hg) under condition of no flow. The preparation was transferred to the stage of an inverted microscope (CK2, Olympus, Lake Success, NY). Attached to the microscope were a CCTV camera (WV-BL200, Panasonic, Secaucus, NJ), video monitor (Panasonic), and a video caliper (VIA-100 K, Boeckeler Instruments, Inc., Tucson, AZ). The organ chamber was connected to a rotary pump (Masterflex, Cole Parmer Instrument Co., Vernon Hills, IL), which continuously circulated oxygenated PSS at 30 ml/min and warmed to 37°C. The pressure within the vessel was then slowly increased to 40 mm Hg. At this pressure we found that KCl gave the maximal constrictor response. Therefore, all the studies were conducted at 40 mm Hg. Internal diameter (resolution of 2 μm) was measured by manually adjusting the video micrometer. After 30 min equilibration, KCl was added to the bath to test vessel viability. Vessels, which failed to constrict more than 30%, were discarded. After washing with PSS, vessels were incubated for 30 min in PSS and then constricted with U46619 ($10^{-8} - 10^{-7}$ M) to 30 – 50% of passive diameter. There was no significant difference in the amount of U46619 required to induce constriction in control and diabetic vessels. Cumulative concentration-response relationships were evaluated for acetylcholine ($10^{-8} - 10^{-4}$ M) using vessels from control and diabetic rats. At the end of each acetylcholine dose response determination sodium nitroprusside ($10^{-4}$ M) was added to determine maximal vasodilation. In a separate set of experiments arterioles were isolated to determine superoxide levels using hydroethidine as previously described.[20]

### Detection of Superoxide

Hydroethidine, an oxidative fluorescent dye was used to evaluate *in situ* levels of superoxide ($O_2^-$) as described previously.[20,21] Hydroethidine is permeable to cells and in the presence of $O_2^-$ is oxidized to fluorescent ethidium bromide, where it is trapped by intercalating with DNA. This method provides sensitive detection of $O_2^-$ *in situ*. Unfixed frozen ring segments were cut into 30-μm-thick sections and placed on glass slides. Hydroethidine ($2 \times 10^{-6}$ M) was topically applied to each tissue section and coverslipped. Slides were incubated in a light protected humidified chamber at 37°C for 30 minutes. Images were obtained with a Bio-Rad MRC-1024 laser scanning confocal microscope equipped with a krypton/argon laser. Fluorescence was detected with a 585-nm long pass filter. Normal and diabetic tissues were processed and imaged in parallel. Laser settings were identical for acquisition of images from normal and diabetic specimens.

### Sciatic Nerve Sorbitol, Fructose and Myo-inositol Content

The left sciatic nerve was removed, desheathed, and weighed for determination of $Na^+/K^+$ ATPase activity and conjugated diene levels (as described below) and sorbitol, fructose and myo-inositol content.[1,18] For the latter determination, tissue samples were boiled for 10 min in water containing $\alpha$-D-methylmannopyranoside as an internal standard and deproteinized with 0.5 ml each of 0.19 M $Ba(OH)_2$ and 0.19 M $ZnSO_4$. Following centrifugation the supernatant was collected, frozen, and lyophilized. Afterwards, the samples were derivatized and intracellular contents determined by gas-liquid chromatography as previously described.[1,18].

### $Na^+/K^+$ ATPase Activity

Total and ouabain-inhibited $Na^+/K^+$ ATPase activities were measured in crude homogenates of sciatic nerve.[1,18] Sciatic nerves were desheathed and homogenized in a polytron, utilizing 3 10 sec bursts, at 4°C in 1 ml of 0.2 M sucrose, 0.02 M Tris-HCl buffer, pH 7.5. The samples were then centrifuged at 100- × g for 10 min at 4°C. An aliquot of the supernatant (50 µl) was added to two cuvettes containing 100 mM NaCl, 10 mM KCl, 2.5 mM $MgCl_2$, 2 mM ethylene glycolbis($\beta$-aminoethyl ether)-$N_1$-N′-tetraacetic acid (EGTA), 1 mM Tris-ATP, 1 mM 3-(cyclohexylammonium) phosphoenolpyruvate, 30 mM imidazole-HCl buffer (pH 7.3), 0.15 mM NADH, 50 µg lactate dehydrogenase, 30 µg pyruvate kinase with or without 1 mM ouabain to inhibit the ouabain-sensitive $Na^+/K^+$ ATPase fraction. After a 20 min stabilization period, the oxidation of NADH was recorded over a 30 min period. The activity was expressed as µmol ADP/g wet weight/h. Each assay was conducted in triplicate.

### Additional Biological Parameters

Lactate/pyruvate ratios for the aorta were determined using perchloric acid extracts of the tissue and high performance liquid chromatography as previously described.[22] Lens glutathione (GSH), serum TBARS and sciatic nerve conjugated diene levels were determined as markers of oxidative stress. Lens glutathione levels were determined according to Lou *et al.*[23] Lens were weighed and homogenized in cold 1 ml of 10% trichloroacetic acid and centrifuged for 15 min at 1000- × g. The supernatant (100 µl) was mixed with 0.89 ml of 1.0 M Tris, pH 8.2, and 0.02 M EDTA. Afterwards, 10 µl of dithionitrobenzene (DTNB) was added and change in absorbance measured at 412 nm. A glutathione standard curve (100 – 500 ng) was performed for each assay. The data were recorded µg/mg wet wt. TBARS level in serum was determined by the method of Mihara *et al.*[24] as modified by Siman and Eriksson.[25] Briefly, 200 µl of serum was boiled in 0.75 ml of phosphoric acid (0.19 M), 0.25 ml thiobarbituric acid (0.42 mM) and 0.3 ml water for 60 min. Afterwards, the samples were precipitated with methanol/NaOH and centrifuged for 5 min. The supernatant was measured fluorometrically at excitation wavelength 532 nm and emission wavelength 553 nm. Standards were prepared from malonyldialdehydbis(dietylacetal) and were treated identically as the serum samples. The data was reported µg/ml serum. Sciatic nerve conjugated diene levels were determined according to the method of Recknagel and Ghoshal[26] as adapted to peripheral nerve tissue by Low and Nicklander.[27] Briefly, a segment of the sciatic nerve was extracted with chloroform and methanol. The lipid extract was evaporated and redissolved in 1 ml cyclohexane. Conjugated diene levels were determined by measuring the absorbance at 233 nm with extraction blanks used as references. An extinction coefficient of $2.52 \times 10^4$ M was used.

### Data Analysis

The results are presented as mean ± SE. Comparisons between the groups (control *vs.* diabetic) for MNCV, EBF, sciatic nerve $Na^+/K^+$ ATPase activity, sciatic nerve sorbitol, fructose and myo-inositol content, serum TBARS, sciatic nerve conjugated diene, aorta lactate/pyruvate ratio and lens glutathione levels were conducted using independent unpaired Student's *t* tests. Dose response curves for acetylcholine for control *vs.* diabetic rats were compared using a two way repeated measures analysis of variance with autoregressive covariance structure using proc mixed program of SAS.[1] Whenever significant interactions were noted for control *vs.* diabetic specific treatment-dose-effects were analyzed using a Bonferoni adjustment. A *p* value of less 0.05 was considered significant. All computations were performed using SAS for Windows version 6.12.

### RESULTS

### Body Weight and Plasma Glucose Levels

Data in Table I show that streptozotocin-induced diabetic rats on average gained less weight than age-matched control rats over the 3–28 day experimental period of this study. At the time of experimentation plasma glucose levels were increased 3-4-fold in diabetic compared to control rats.

### $Na^+/K^+$ ATPase Activity and Sorbitol, Fructose and Myo-inositol Content of the Sciatic Nerve

It has been reported that $Na^+/K^+$ ATPase activity is decreased in the sciatic nerve of diabetic rats and this may be a contributing factor to the slowing of motor nerve conduction velocity.[28–30] Moreover, it has been reported that the sorbitol content of the sciatic nerve is increased in diabetic rats accompanied by a reciprocal decrease in the myo-inositol content.[18,31,32] These changes have also been linked to nerve dysfunction in diabetic rats.[18,31,32] In the present study we examined the time course for the development of these changes in the sciatic nerve of diabetic rats in relation to slowing of motor nerve conduction velocity and changes in vascular function. Data in Table II demonstrate that ouabain-sensitive $Na^+/K^+$ ATPase activity in the sciatic nerve from diabetic rats is decreased by about 10% following 1 week of diabetes and is significantly reduced after 4 weeks. The fructose and sorbitol content of the sciatic nerve is significantly increased after 3 and 7 days of diabetes, respectively. In contrast, the myo-inositol content is significantly decreased following 1 week of diabetes and after 4 weeks of diabetes is reduced by about 65%.

### Lens Glutathione Level and Serum TBARS

As a measurement of oxidative stress we determined lens glutathione level and serum TBARS in streptozotocin-induced diabetic rats following 3–28 days of diabetes. Data in Table III demonstrate that lens glutathione levels are significantly decreased following 1 week of diabetes and serum TBARS are increased

TABLE I Change in body weight and blood glucose levels

| Animal | Change in body weight (g/week) | Blood glucose mg/dl |
|---|---|---|
| Control (*n*=11)* | 35 ± 6 | 105 ± 8 |
| Diabetic (Days) | | |
| 3 (*n* = 7) | 4 ± 4 | 348 ± 15⁺ |
| 7 (*n* = 6) | 22 ± 6 | 411 ± 23⁺ |
| 14 (*n* = 7) | 17 ± 7 | 430 ± 26⁺ |
| 21 (*n* = 7) | 4 ± 4 | 448 ± 10⁺ |
| 28 (*n* = 7) | 1 ± 3 | 458 ± 24⁺ |

Data are means ± S.E.M. The number of experimental observations is indicated by parenthesis.
*Control rats were studied at one and three weeks.
⁺$p < 0.05$ *vs.* control.

TABLE II Changes in $Na^+/K^+$ ATPase activity and sorbitol, fructose and myo-inositol content in the sciatic nerve of streptozotocin-induced diabetic rats

| Animal | Ouabain-sensitive $Na^+/K^+$ ATPase (μmol ADP/g wet wt/h) | Content (nmol/mg wet wt) | | |
|---|---|---|---|---|
| | | Sorbitol | Fructose | Myo-inositol |
| Control | 235.0 ± 16.4 | 0.2 ± 0.1 | 0.4 ± 0.1 | 11.0 ± 1.4 |
| Diabetic (Days) | | | | |
| 3 (7) | 256.7 ± 31.1 | 0.5 ± 0.2 | 1.7 ± 0.2$^+$ | 8.7 ± 1.4 |
| 7 (6) | 209.1 ± 34.0 | 1.0 ± 0.3$^+$ | 2.7 ± 1.0$^+$ | 6.1 ± 1.2$^+$ |
| 14 (7) | 195.5 ± 25.0 | 1.0 ± 0.1$^+$ | 3.1 ± 0.4$^+$ | 5.5 ± 1.2$^+$ |
| 21 (7) | 193.7 ± 15.0 | 1.1 ± 0.3$^+$ | 3.6 ± 0.5$^+$ | 5.1 ± 0.5$^+$ |
| 28 (7) | 181.0 ± 16.4$^+$ | 1.2 ± 0.2$^+$ | 3.2 ± 0.6$^+$ | 3.7 ± 0.4$^+$ |

Data are mean ± S.E.M. The number of experimental observations is indicated by parenthesis.
$^+p < 0.05$ *vs.* control.

TABLE III Lens glutathione level and serum TBARS in streptozotocin-induced diabetic rats

| Animal | Glutathione (μg/mg wet wt) | TBARS (μg/ml serum) |
|---|---|---|
| Control | 1.24 ± 0.22 | 11.9 ± 2.7 |
| Diabetic (Days) | | |
| 3 (7) | 0.79 ± 0.13 | 14.7 ± 3.1 |
| 7 (6) | 0.26 ± 0.06$^+$ | 14.1 ± 3.6 |
| 14 (7) | 0.24 ± 0.05$^+$ | 16.7 ± 3.1 |
| 21 (7) | 0.18 ± 0.01$^+$ | 23.9 ± 3.2$^+$ |
| 28 (7) | 0.28 ± 0.04$^+$ | 23.9 ± 3.4$^+$ |

Data are means ± S.E.M. The number of experimental observations is indicated by parenthesis.
$^+p < 0.05$ *vs.* control.

reaching significance after 3 weeks of diabetes. We also examined conjugated diene levels in the sciatic nerve of diabetic rats. In these studies levels of conjugated dienes were increased significantly by about 2–3 fold as early as 1 week after the induction of diabetes (data not shown).

### Motor Nerve Conduction Velocity and Endoneural Blood Flow in Diabetic Rats

Data in Table IV demonstrate that motor nerve conduction velocity was significantly decreased after 2 weeks of diabetes. Endoneural blood flow reported as nutritive flow (ml/min/100 g) and conductance (ml/min/100 g/mm Hg) were significantly reduced by about 50% following 3 days of diabetes compared to control rats and remained reduced after 4 weeks of diabetes (Tab. IV). In these studies the mean arterial blood pressure was not significantly different between the control and diabetic rats (data not shown).

TABLE IV Time course for the development of motor nerve conduction and endoneural blood flow defects in the streptozotocin-induced diabetic rat

| Animal | MNCV m/sec | Endoneural blood flow | |
|---|---|---|---|
| | | Nutritive (ml/min/100 g) | Conductance (ml/min/100 g/mm Hg) |
| Control (11) | 52.4 ± 2.5 | 18.2 ± 2.1 | 0.154 ± 0.016 |
| Diabetic (Days) | | | |
| 3 (7) | 49.6 ± 1.9 | 9.6 ± 1.5$^+$ | 0.084 ± 0.012$^+$ |
| 7 (6) | 52.6 ± 3.9 | 8.6 ± 2.7$^+$ | 0.070 ± 0.019$^+$ |
| 14 (7) | 41.3 ± 1.7$^+$ | 10.6 ± 2.4$^+$ | 0.097 ± 0.011$^+$ |
| 21 (7) | 38.6 ± 1.6$^+$ | 9.9 ± 1.5$^+$ | 0.087 ± 0.012$^+$ |
| 28 (7) | 41.6 ± 1.3$^+$ | 9.3 ± 1.1$^+$ | 0.084 ± 0.011$^+$ |

Data are means ± S.E.M. The number of experimental observations is indicated by parenthesis.
$^+p < 0.05$ *vs.* control.

### Analysis of Superoxide Levels

Data in Figure 1 show that after 2 weeks of diabetes superoxide ($O_2^-$) levels as measured by hydroethidine fluorescence are increased in arterioles that provide circulation to the region of the sciatic nerve compared to normal vessels. The increase in $O_2^-$ levels was observed in endothelial cells as well as in the smooth muscle and adventitial cells. Analysis of $O_2^-$ levels in vessels from 3 and 7-day diabetic rats suggest that the increase in $O_2^-$ in diabetic rats was a gradual process and appeared to approach maximum after 2 weeks of diabetes. No further increase in $O_2^-$ levels was apparent in vessels after 3 and 4 weeks of diabetes. As a control, preincubating vessels from diabetic rats with superoxide dismutase quenched the hydroethidine fluorescence (data not shown).

### Arteriolar Vascular Reactivity in Diabetic Rats

Stimulated changes in vascular diameter were measured *in vitro* by application of acetylcholine.[1] Baseline diameter of vessels from control and diabetic rats was similar and the vessels were constricted to a similar degree with U46619 (10 – 100 μM). Acetylcholine produced a concentration-dependent vasodilation (endothelium-dependent) in arterioles that provide circulation to the region of the sciatic nerve (maximum dilation = 93 ± 2%) (Fig. 2, Tab. V). At 3 days, the vascular responses to acetylcholine were similar in diabetic and normal rats (Fig. 2, Tab. V). At 7 days, vascular relaxation to acetylcholine was impaired in diabetic rats at a lower dose of acetylcholine ($1 \times 10^{-7}$ M), however maximal relaxation was unchanged from normal animals (Fig. 2, Tab. V). After 14 days, vascular relaxation in diabetic rats was significantly less at all doses of acetylcholine compared 'to normal rats (Fig. 2, Tab. V). Maximal relaxation to acetylcholine was significantly impaired in vessels from 2-week diabetic (62 ± 10%) compared to normal (93 ± 2%) rats (Tab. V). Similar results were also obtained at 3 and 4 weeks of diabetes (Tab. V). In contrast, maximal vasodilation induced by sodium nitroprusside (endothelium-independent) was

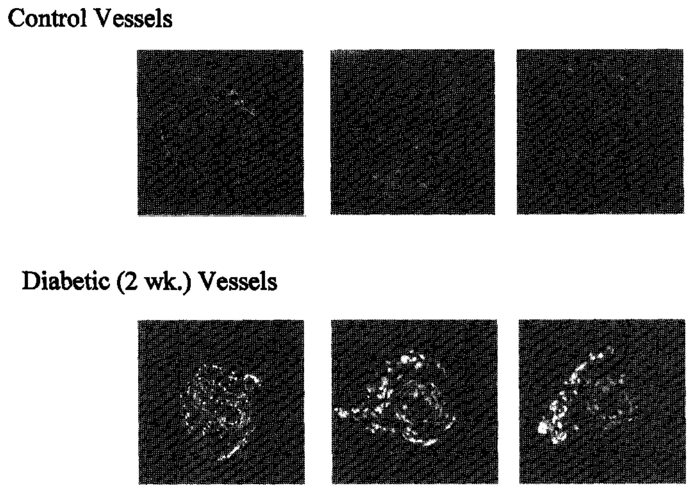

FIGURE 1 Detection of superoxide levels in arterioles from control and 2-week diabetic rats. Fluorescent photomicrographs of confocal microscopic sections of arterioles that provide circulation to the region of the sciatic nerve from three individual control and 2-week streptozotocin-induced diabetic rats were examined on three separate days. Arterioles were labeled with the oxidative dye hydroethidine as described in the Methods section. Recording of fluorescent were taken at identical laser and photomultiplier settings for both control and diabetic rats.

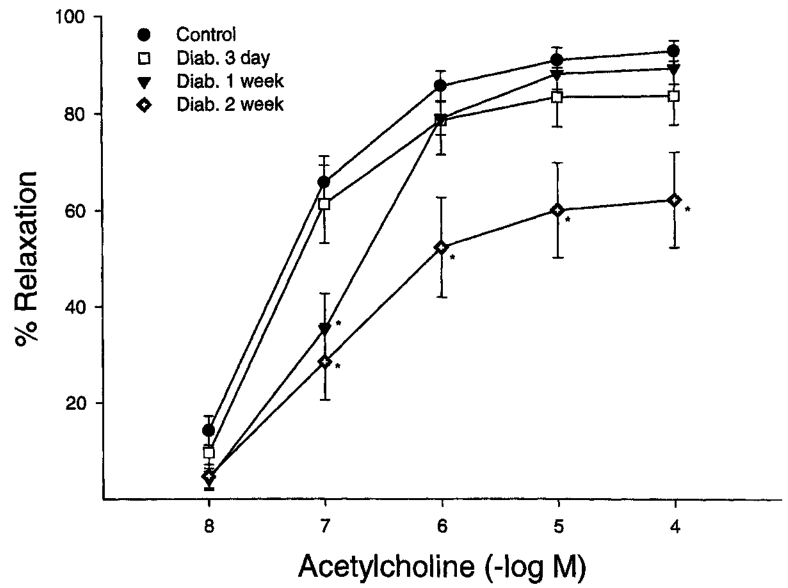

FIGURE 2 Acetylcholine-induced endothelium dependent relaxation of arterioles adjacent to the sciatic nerve in control and diabetic rats. For these studies control rats and rats with diabetes for 3, 7 and 14 days were used. Pressurized arterioles were constricted with U46619 (30–50%) and incremental doses of acetylcholine were added to the bathing solution while recording steady state vessel diameter. The number of experimental animals used in these studies was the same as noted in Table I. The * denotes that the response to acetylcholine was significantly attenuated ($p < 0.05$) in the diabetic rat.

TABLE V Relaxation of arterioles adjacent to the sciatic nerve from control and streptozotocin-induced diabetic rats: Effect on acetylcholine-induced $ED_{50}$ and maximum response and sodium nitroprusside maximal dilation

| | Acetylcholine | | SNP |
|---|---|---|---|
| Animal | $ED_{50}$(nM) | Max % relaxation | (% relaxation) |
| Control (11) | $56 \pm 15$ | $93 \pm 2$ | $95 \pm 2$ |
| Diabetic (Days) | | | |
| 3 (10) | $71 \pm 32$ | $84 \pm 6$ | $88 \pm 6$ |
| 7 (9) | $190 \pm 42^{+}$ | $89 \pm 3$ | $94 \pm 3$ |
| 14 (9) | $328 \pm 155^{+}$ | $62 \pm 10^{+}$ | $85 \pm 7$ |
| 21 (10) | $258 \pm 103^{+}$ | $74 \pm 5^{+}$ | $85 \pm 4$ |
| 28 (10) | $375 \pm 143^{+}$ | $73 \pm 8^{+}$ | $88 \pm 6$ |

Data are means ± S.E.M. The number of experimental observations is indicated by parenthesis.
$^{+}p < 0.05$ *vs.* control.

not significantly affected by diabetes in these vessels (Tab. V).

### DISCUSSION

The results from these studies demonstrate that diabetes-induced decrease in endoneural blood flow and impairment of acetylcholine-induced vasodilation of arterioles that provide circulation to the region of the sciatic nerve precedes slowing of motor nerve conduction and decrease in $Na^{+}/K^{+}$ ATPase activity in the sciatic nerve. Moreover, our studies show that the generation of superoxide in vasculature that provides circulation to the region of the sciatic nerve accompanies the diabetes-induced impairment in vasodilation. Other markers of oxidative stress are also increased during this period in the streptozotocin-induced diabetic rat model. These studies suggest that vascular dysfunction may be the major factor contributing to the development of diabetic neuropathy.

Studies by Greene and co-workers have shown that a reduction in $Na^+/K^+$ ATPase activity in the sciatic nerve of diabetic rats may be linked to an increase in the sorbitol content and reduction in myo-inositol levels in the sciatic nerve.[28-31] These disturbances have been proposed to be partially responsible for the slowing of nerve conduction velocity in the diabetic rat.[18,28-32] Our studies show that the reciprocal changes in the level of sorbitol and myo-inositol in the sciatic nerve of the diabetic rat occurs early in diabetes and precedes the reduction in $Na^+/K^+$ ATPase activity. However, in our studies slowing of motor nerve conduction velocity occurs prior to the reduction in $Na^+/K^+$ ATPase activity in the sciatic nerve. This suggests that the early change in nerve conduction velocity in the streptozotocin-induced diabetic rat is not due to a reduction in $Na^+/K^+$ ATPase activity. It is possible that the reduction in $Na^+/K^+$ ATPase activity in the sciatic nerve enhances neural dysfunction but that the initial defect responsible for slowing of motor nerve conduction velocity is caused by vascular changes.

Cameron *et al.*, showed that nerve blood flow was reduced by about 40% as early as 1 week after the induction of diabetes.[2] In the same study they also showed that acute hyperglycemia maintained over the period required to measure nerve blood flow (2–4 h) caused a 50% reduction in blood flow.[2] Stevens *et al.*, using laser Doppler flux to measure nerve blood flow found that flow was variable during the first two days following streptozotocin injection, was reduced by 20% after four days of diabetes and fell steadily until reaching a plateau at 40% of control values after 4 weeks of diabetes.[33] Similar results were obtained in our studies. We found that endoneural blood flow was significantly reduced by about 50% three days after the induction of diabetes and remained decreased for the 4-week period of the study.

The determination of the sequential development of vascular dysfunction in arterioles that provide circulation to the region of the sciatic nerve demonstrates that impairment of endothelial-dependent vasodilation occurs early in diabetes. One week after the induction of diabetes, vasodilation in response to a low dose of acetylcholine ($10^{-7}$ M) was significantly decreased. However, maximum vasodilation in response to acetylcholine was not significantly reduced at this time, although two weeks after the induction of diabetes vasodilation in response to all doses of acetylcholine was significantly reduced and maximum vasodilation was decreased by about 30%. This effect remained relatively consistent for up to 4 weeks after the induction of diabetes. This study is among the first to show the sequential development of diabetes-induced changes in endoneural blood flow, vascular reactivity and motor nerve conduction velocity. It clearly demonstrates that diabetes-induced impairment of endothelial-dependent vasodilation by arterioles providing circulation to the region of the sciatic nerve precedes the slowing of motor nerve conduction velocity. This study also implies that impairment of acetylcholine-induced vasodilation is preceded by the reduction in endoneural blood flow suggesting that diabetes-induced impairment of endothelial dependent vasodilation in this vascular bed is not initially related to the defect in endoneural blood flow. One possible explanation for this finding is that the initial reduction in endoneural blood flow is due to impaired vasodilation of vessels within the nerve, which may occur earlier in diabetes.

Diabetes-induced impairment of vascular reactivity is a common feature in both conduit and resistance arteries from diabetic animal models.[34] Impaired endothelium-dependent vasodilation in conduit and resistance arteries in patients with insulin-dependent diabetes has also been reported.[35,36] However, our previous studies are among the first to show that endothelium-dependent vasodilation by arterioles that provide circulation to the sciatic nerve is altered by diabetes.[1] The mechanism responsible for diabetes-induced impairment of vascular reactivity is unknown. In the aorta, diabetes causes the endothelium to increase the production of both superoxide and hydrogen peroxide leading to enhanced intracellular production of hydroxyl radicals.[37] Studies by Pieper and colleagues have suggested that the generation of hydroxyl radicals may mediate the damage caused by diabetes to the endothelium of the aorta.[34, 37–40] Other studies have suggested that endothelial dysfunction in smaller vessels may be mediated by the increased production of free radicals and of prostaglandin endoperoxides.[14] In resistance-level arterioles of the mesenteric circulation diabetes causes a reduction in endothelium-dependent vasodilation.[9, 41] Heygate *et al.*, reported that pretreatment of mesenteric arteries from diabetic rats with indomethacin significantly improved acetylcholine-induced vasodilation suggesting a role for a cyclooxygenase-derived vasoconstrictor prostanoids in mediating diabetes-induced vascular dysfunction in these arteries.[9] In addition, Rodriguez-Manas *et al.*, reported that pretreatment of mesenteric arteries from diabetic rats with superoxide dismutase improved acetylcholine responsiveness suggesting that the increased production of superoxide anions in the vascular wall of mesenteric arteries may also mediate vascular dysfunction.[11] Mayhan reported similar results using basilar arteries from diabetic rats.[42] In the latter study topical application of superoxide dismutase partially restored acetylcholine-induced vasodilation of the basilar artery towards that observed in nondiabetic rats suggesting that impaired vasodilation may be related in part to enhanced release of oxygen-derived free radicals.[42] Other studies support a role for the production of oxygen radicals in diabetic neuropathy. Studies with diabetic rats incorporating the use of anti-oxidant treatment have been shown to prevent peripheral nerve dysfunction.[43–46]

In our studies we have shown that acetylcholine-induced vasodilation of arterioles that provide circulation to the region of the sciatic nerve is mediated by at least two mechanisms involving the production of nitric oxide (NO) and endothelium-derived hyperpolarizing factor (EDHF).[1] Therefore, impairment of endothelium-dependent vasodilation by diabetes in these vessels may be mediated by conditions that inhibit the production or bioactivity of either or both of these vasodilators. The present study demonstrates that diabetes causes the production/accumulation of superoxide in these vessels. As discussed above, the production of oxygen radicals has been associated with impairment of endothelium-dependent vasodilation in both conduit and resistance arteries. Presently, we do not know whether the generation of superoxide or the secondary production of hydrogen peroxide or hydroxyl radicals by these vessels might contribute to diabetes-induced vascular dysfunction.

In summary, these studies demonstrate that endothelium-dependent vasodilation of arterioles that provide circulation to the region of the sciatic nerve is impaired early in diabetes and along with reduction in endoneural blood flow precedes the slowing of motor nerve conduction velocity and reduction of sciatic nerve $Na^+/K^+$ ATPase activity. In addition the generation and accumulation of superoxide by these arterioles coincides with the impairment of vasodilation. These results suggest that vascular dysfunction that may be caused by the production of free oxygen radicals may be the major factor contributing to the early development of diabetic neuropathy.

### *Acknowledgments*

This work was supported by a National Institute of Diabetes and Digestive and Kidney Diseases Grant DK-25295, by a grant from the National

Institute of Diabetes and Digestive and Kidney Diseases DK-58005, by a Diabetes Center Grant from the Veterans Affairs and International Juvenile Diabetes Foundation, and by a research grant from the American Diabetes Association. The authors would like to express their appreciation to Ms. Pam Tompkins for her assistance in the measurement of superoxide levels.

### References

[1] Terata, K., Coppey, L. J., Davidson, E. P., Dunlap, J. A., Gutterman, D. D. and Yorek, M. A. (1999). Acetylcholine-induced arteriolar dilation is reduced in streptozotocin-induced diabetic rats with motor nerve dysfunction, *British J. Pharm.*, **128**, 837–843.

[2] Cameron, N. E., Cotter, M. A. and Low, P. A. (1991). Nerve blood flow in early experimental diabetes in rats: relation to conduction deficits, *Am. J. Physiol.*, **261**, E1–E8.

[3] Wright, R. A. and Nukada, H. (1994). Vascular and metabolic factors in the pathogenesis of experimental diabetic neuropathy in mature rats, *Brain*, **117**, 1395–1407.

[4] Tesfamariam, B., Brown, M. L. and Cohen, R. A. (1991). Elevated glucose impairs endothelium-dependent relaxation by activating protein kinase C, *J. Clin. Invest.*, **87**, 1643–1648.

[5] Tesfamariam, B. and Cohen, R. A. (1992). Free radicals mediate endothelial cell dysfunction caused by elevated glucose, *Am. J. Physiol.*, **263**, H321–H326.

[6] Tesfamariam, B., Palacino, J. J., Weisbrod, R. M. and Cohen, R. A. (1993). Aldose reductase inhibition restores endothelial cell function in diabetic rabbit aorta, *J. Cardiovasc. Pharm.*, **21**, 205–211.

[7] Dorigo, P., Fraccarollo, D., Santostasi, G. and Maragno, I. (1997). Impairment of endothelium-dependent but not of endothelium-independent dilatation in guinea-pig aorta rings incubated in the presence of elevated glucose, *Br. J. Pharmacol.*, **121**, 972–976.

[8] Taylor, P. D. and Poston, L. (1994). The effect of hyperglycemia on function of rat isolated mesenteric resistance artery, *Br. J. Pharmacol.*, **113**, 801–808.

[9] Heygate, K. M., Lawrence, I. G., Bennett, M. A. and Thurston, H. (1995). Impaired endothelium-dependent relaxation in isolated resistance arteries of spontaneously diabetic rats, *Br. J. Pharmacol.*, **116**, 3251–3259.

[10] Tribe, R. M., Thomas, C. R. and Poston, L. (1998). Flow-induced dilatation in isolated resistance arteries from control and streptozotocin-diabetic rats, *Diabetologia*, **41**, 34–39.

[11] Rodriguez-Manas, L., Angulo, J., Peiro, C., Llergo, J. L., Sanchez-Ferrer, A., Lopez-Doriga, P. and Sanchez-Ferrer, C. F. (1998). Endothelial dysfunction and metabolic control in streptozotocin-induced diabetic rats, *Br. J. Pharmacol.*, **123**, 1495–1502.

[12] Fujii, K., Heistad, D. D. and Faraci, F. M. (1992). Effect of diabetes mellitus on flow-mediated and endothelium-dependent dilatation of the rat basilar artery, *Stroke*, **23**, 1494–1498.

[13] Mayhan, W. G., Didion, S. P. and Patel, K. P. (1996). L-Arginine does not restore dilatation of the basilar artery during diabetes mellitus, *J. Cerebral Blood Flow Met.*, **16**, 500–506.

[14] Dai, F., Diederich, A., Skopec, J. and Diederich, D. (1993). Diabetes-induced endothelial dysfunction in streptozotocin-treated rats: role of prostaglandin endoperoxides and free radicals, *J. Am. Soc. Nephrol.*, **4**, 1327–1336.

[15] Matsunaga, T., Okumura, K., Ishizaka, H., Tsunoda, R., Tayama, S., Tabuchi, T. and Yasue, H. (1996). Impairment of coronary blood flow regulation by endothelium-derived nitric oxide in dogs with alloxan-induced diabetes, *J. Cardiovasc. Pharm.*, **28**, 60–67.

[16] Koltai, M. Z., Hadhazy, P., Posa, I., Kocsis, E., Winkler, G., Rosen, P. and Pogatsa, G. (1997). Characteristics of coronary endothelial dysfunction in experimental diabetes, *Cardiovasc. Res.*, **34**, 157–163.

[17] Kuo, L., Chilian, W. M. and Davis, M. J. (1990). Coronary arteriolar myogenic response is independent of endothelium, *Circ. Res.*, **66**, 860–866.

[18] Yorek, M. A., Wiese, T. J., Davidson, E. P., Dunlap, J. A., Stefani, M. R., Conner, C. E., Lattimer, S. A., Kamijo, M., Greene, D. A. and Sima, A. A. F. (1993). Reduced motor nerve conduction velocity and $Na^+$-$K^+$-ATPase activity in rats maintained on L-fucose diet, *Diabetes*, **42**, 1401–1406.

[19] Cameron, N. E., Cotter, M. A., Basso, M. and Hohman, T. C. (1997). Comparison of the effects of inhibitors of aldose reductase and sorbitol dehydrogenase on neurovascular function, nerve conduction and tissue polyol pathway metabolites in streptozotocin-diabetic rats, *Diabetologia*, **40**, 271–281.

[20] Lund, D. D., Faraci, F. M., Miller, F. J. Jr. and Heistad, D. D. (2000). Gene transfer of endothelial nitric oxide synthase improves relaxation of carotid arteries from diabetic rabbits, *Circulation*, **101**, 1027–1033.

[21] Miller, F. J., Gutterman, D. D., Rios, C. D., Heistad, D. D. and Davidson, B. L. (1998). Superoxide production in vascular smooth muscle contributes to oxidative stress and impaired relaxation in atherosclerosis, *Circ. Res.*, **82**, 1298–1305.

[22] Hallstrom, A., Carlsson, A., Hillered, L. and Ungerstedt, U. (1989). Simultaneous determination of lactate, pyruvate, and ascorbate in microdialysis samples from rat brain, blood, fat, and muscle using high-performance liquid chromatography, *J. Pharm. Methods*, **22**, 113–124.

[23] Lou, M. F., Dickerson, J. E. Jr., Garadi, R. and York, B. M. Jr. (1988). Glutathione depletion in the lens of galactosemic and diabetic rats, *Exp. Eye Res.*, **46**, 517–530.

[24] Mihara, M., Uchiyama, M. and Fukuzama, K. (1980). Thiobarbituric acid value of fresh homogenate of rat as a parameter of lipid peroxidation in aging, CC14 intoxication, and vitamin E deficiency, *Biochem. Med.*, **23**, 302–311.

[25] Siman, C. M. and Eriksson, U. J. (1997). Vitamin C supplementation of the maternal diet reduces the rate of malformation in the offspring of diabetic rats, *Diabetologia*, **40**, 1416–1424.

[26] Recknagel, R. O. and Ghoshal, A. K. (1966). Lipoperoxidation as a vector in carbon tetrachloride hepatotoxicity, *Lab. Invest.*, **15**, 132–145.

[27] Low, P. A. and Nickander, K. K. (1991). Oxygen free radical effects in sciatic nerve in experimental diabetes, *Diabetes*, **40**, 873–877.
[28] Greene, D. A., Yagihashi, S., Lattimer, S. A. and Sima, A. A. F. (1984). Nerve $Na^+$-$K^+$-ATPase, conduction, and myo-inositol in the insulin-deficient BB rat, *Am. J. Physiol.*, **247**, E534–E539.
[29] Greene, D. A. and Lattimer, S. A. (1984). Impaired energy utilization and Na-K-ATPase in diabetic peripheral nerve, *Am. J. Physiol.*, **246**, E311–E318.
[30] Greene, D. A., Lattimer, S. A. and Sima, A. A. F. (1988). Are disturbances of sorbitol, phosphoinositide, and $Na^+$-$K^+$-ATPase regulation involved in pathogenesis of diabetic neuropathy? *Diabetes*, **37**, 688–693.
[31] Greene, D. A., Sima, A. A. F., Stevens, M. J., Feldman, E. L., Killen, P. D., Henry, D. N., Thomas, T., Dananberg, J. and Lattimer, S. A. (1993). Aldose reductase inhibitors: an approach to the treatment of diabetic nerve damage, *Diabetes/Metabolism Rev.*, **9**, 189–217.
[32] Sima, A. A. F. (1995). Aldose reductase inhibitors in the treatment of diabetic complications, *Int. J. Diabetes*, **3**, 158–167.
[33] Stevens, E. J., Carrington, A. L., and Tomlinson, D. R. (1994). Nerve ischaemia in diabetic rats: time-course of development, effect of insulin treatment plus comparison of streptozotocin and BB models, *Diabetologia*, **37**, 43–48.
[34] Pieper, G. M. (1998). Review of alterations in endothelial nitric oxide production in diabetes: protective role of arginine on endothelial dysfunction, *Hypertension*, **31**, 1047–1060.
[35] Johnstone, M. T., Creager, S. J., Scales, K. M., Cusco, J. A., Lee, B. K. and Creager, M. A. (1993). Impaired endothelium-dependent vasodilation in patients with insulin-dependent diabetes mellitus, *Circulation*, **88**, 2510–2516.
[36] Lekakis, J., Papamichael, C., Anatasiou, H., Alevizaki, M., Desses, N., Souvatzoglou, A., Stamatelopoulos, S. and Koutras, D. A. (1997). *Endothelial dysfunction of conduit arteries in insulin-dependent diabetes mellitus without microalbuminuria*, **34**, 164–168.
[37] Pieper, G. M., Langenstroer, P. and Siebeneich, W. (1997). Diabetic-induced endothelial dysfunction in rat aorta: role of hydroxyl radicals, *Cardiovascular Res.*, **34**, 145–156.
[38] Pieper, G. M., Langenstroer, P. and Gross, G. J. (1993). Hydroxyl radicals mediate injury to endothelium-dependent relaxation in diabetic rat, *Mole. Cell. Biochem.*, **122**, 139–145.
[39] Pieper, G. M. and Siebeneich, W. (1997). Diabetes-induced endothelial dysfunction is prevented by long-term treatment with the modified iron chelator, hydroxymethyl starch conjugated-deferoxamine, *J. Card. Pharm.*, **30**, 734–738.
[40] Pieper, G. M. and Siebeneich, W. (1998). Oral administration of the antioxidant, N-acetylcysteine, abrogates diabetes-induced endothelial dysfunction, *J. Card. Pharm.*, **32**, 101–105.
[41] Taylor, P. D., Graves, J. E. and Poston, L. (1995). Selective impairment of acetylcholine-mediated endothelium-dependent relaxation in isolated resistance arteries of the streptozotocin-induced diabetic rat, *Clin. Sci.*, **88**, 519–524.
[42] Mayhan, W. G. (1997). Superoxide dismutase partially restores impaired dilation of the basilar artery during diabetes mellitus, *Brain Res.*, **760**, 204–209.
[43] Cameron, N. E., Cotter, M. A. and Maxfield, E. K. (1993). Anti-oxidant treatment prevents the development of peripheral nerve dysfunction in streptozotocin-diabetic rats, *Diabetologia*, **36**, 299–304.
[44] Cameron, N. E., Cotter, M. A., Archibald, V., Dines, K. C. and Maxfield, E. K. (1994). Anti-oxidant and pro-oxidant effects on nerve conduction velocity, endoneural blood flow and oxygen tension in non-diabetic and streptozotocin-diabetic rats, *Diabetologia*, **37**, 449–459.
[45] Keegan, A., Cotter, M. A. and Cameron, N. E. (1999). Effects of diabetes and treatment with the antioxidant $\alpha$-lipoic acid on endothelial and neurogenic responses of corpus cavernosum in rats, *Diabetologia*, **42**, 343–350.
[46] Cameron, N. E. and Cotter, M. A. (1999). Effects of antioxidants on nerve and vascular dysfunction in experimental diabetes, *Diabetes Res. Clin. Practice*, **45**, 137–146.

